# Research on the design of serious illness insurance scheme in Shanghai based on micro-simulation

**DOI:** 10.1186/s12913-022-07783-z

**Published:** 2022-03-25

**Authors:** Yang Li, Guangfeng Duan, Linping Xiong

**Affiliations:** Department of Health Service, Naval Medical University, Shanghai, 200433 China

**Keywords:** Serious illness medical insurance system, Micro-simulation, Urban and rural residents

## Abstract

**Background:**

Urban and rural residents’ basic medical insurance (URRBMI) is an institutional arrangement for rural residents and unemployed urban residents in China. The serious illness medical insurance system (SIMIS) was established to provide additional medical cover. At present, the SIMIS payment method in China is based on large expenses, and only a few areas, such as Shanghai, pay according to the treatment of serious diseases. This study aims to simulate and analyse the effect of the two payment methods on SIMIS in Shanghai.

**Methods:**

We developed a micro-simulation model to predict the number and characteristics of SIMIS participants among urban and rural residents in Shanghai and to simulate the process of medical treatment, medical consumption, and medical insurance payments for each insured person from 2020 to 2025. We then summarised and analysed the payment compensation effect, and compared it with Shanghai’s current policies.

**Results:**

The payment of SIMIS according to high expenses, the total medical expenses of seriously ill patients show an increasing trend, with an average annual growth rate of 3.56%. The URRBMI fund payment covers 56%–58% of total medical expenses, and the SIMIS fund covers 5%–7% of the total medical expenses. Both cover 62%–63% of total medical expenses. Self-payment under SIMIS covers 22%–23% of the total medical expenses, total self-payment covers 14%–15% of the total medical expenses, and the medical expenses borne by individuals cover 36%–38% of the total medical expenses.The fund expenditure is 213 million yuan and average annual cost borne by individual patients ranges from 40 000 to 60 000 yuan.

**Conclusions:**

The policy of designing SIMIS according to national guidelines does not meet the development needs of Shanghai. Shanghai should take the current policy of paying compensation according to the treatment of serious illness as the policy basis, consider the security needs of patients with large medical expenses outside the scope of protection, and adjust policies appropriately to prevent poverty caused by illness.

## Background

After years of development, China’s medical security system has established urban employees’ basic medical insurance (UEBMI) and urban and rural residents’ basic medical insurance (URRBMI). China’s medical security system has also achieved full coverage [1]. There is an institutional arrangement for rural and urban residents who do not participate in UEBMI. Although the system alleviates the burden of residents’ medical expenses, its guarantee is limited due to the high cost of medical expenses [2, 3]. To further improve URRBMI, China established a serious illness medical insurance system (SIMIS) [4]. There are two ways to compensate for medical expenses: the first is to pay for large expenses in proportion to personal contributions after the payment of URRBMI. The second payment method, which is for the treatment of serious diseases, is paid in proportion to personal contributions after the payment of URRBMI. Shanghai’s SIMIS is based on the treatment of serious illnesses [5].

In this study, we attempt to design a SIMIS based on large expense payments on the basis of URRBMI with reference to national guidelines, and to analyse the effect of the designed scheme in Shanghai by using micro-simulation technology. Then, we compare and analyse the implementation effect with the current serious illness insurance policy in Shanghai and discuss the feasible practices of SIMIS in Shanghai.

## Methods

The micro-simulation model is a computer program designed to use individual-level data. It is a special type of simulation technology. Through a simulation of each individual’s relevant behaviour (such as medical behaviour), it implements relevant policies on individuals, estimates and predicts the future development trend of a group under certain conditions, judges the impact of policy adjustments on individual distributions, and infers and synthesises the macro effect of policy implementation [6–9].

The realisation of the micro-simulation model depends on the quality of the data files. The development of database technology directly affects the accuracy, efficiency, and practicability of the model. The idea of modelling is: sampling the individuals to be studied to obtain a micro database, that is, to establish the environment for the model simulation. Then the simulation model is constructed according to the behaviour of individuals in various systems of society, that is, the main behaviour patterns of individuals in the model are constructed. Computer technology is used to simulate the changes in individual characteristics in response to changes in the relevant policy parameters and characteristics, that is, the simulation results are obtained through the operation of the model. Based on the statistics, inferences, analyses, and the synthesis of characteristic indicators, the impact of policy adjustments on individuals on a micro level is obtained, the effect of policy implementation at all levels is analysed, and the simulation results are summarised and analysed.

The application process is as follows. (1) Based on the change law of the relevant characteristics of insured individuals, the number and characteristics of the SIMIS insured population from 2020 to 2025 are estimated. (2) Based on the national guiding policy on serious illness insurance, this study designs the payment policy of SIMIS in Shanghai according to large expenses, and constructs a micro-simulation model. (3) The micro database is used to determine the medical treatment distribution, medical consumption distribution, basic medical insurance payment proportion, and serious illness insurance payment proportion of patients with large expenses. A random method is used to simulate the medical consumption and medical insurance payment process of each insured person, and to summarise and analyse the effect after the implementation of a serious illness insurance policy.

### Data

The individual data (*n* = 381,363) of medical insurance payments of 2% of the insured population of Shanghai from 2011 to 2016 were randomly selected from the population with basic medical insurance, covering UEBMI and URRBMI. The treatment categories were outpatients and inpatients. The database provides the following information: (1) identification number, (2) age, (3) gender, (4) insurance type: URRBMI or UEBMI, (5) diagnosis of inpatients, (6) total medical expenses, (7) URRBMI or UEBMI fund payment expenses, (8) self-payment expenses under the URRBMI or UEBMI, and (9) total self-payment expenses. The data include information on all personal characteristics and medical insurance payments.

### Scheme design and model construction

#### Payment scheme design

This study is based on the guidelines of SIMIS in China that determine serious illness in patients based on large expenses. The setting of the threshold payment of SIMIS in most parts of China is mainly based on disposable income per capita per year before the implementation of the policy. This study assumes that the SIMIS policy in Shanghai was implemented in 2016. In 2015, the per capita disposable income of rural family residents in Shanghai was 25 520 yuan. Based on this, the threshold payment for SIMIS was 25 000 yuan. In view of the high level of economic development in Shanghai, there is no ceiling limit for the total SIMIS payment and the payment proportion is set at 60% according to the national policy guidelines [1].

**Fig. 1:**
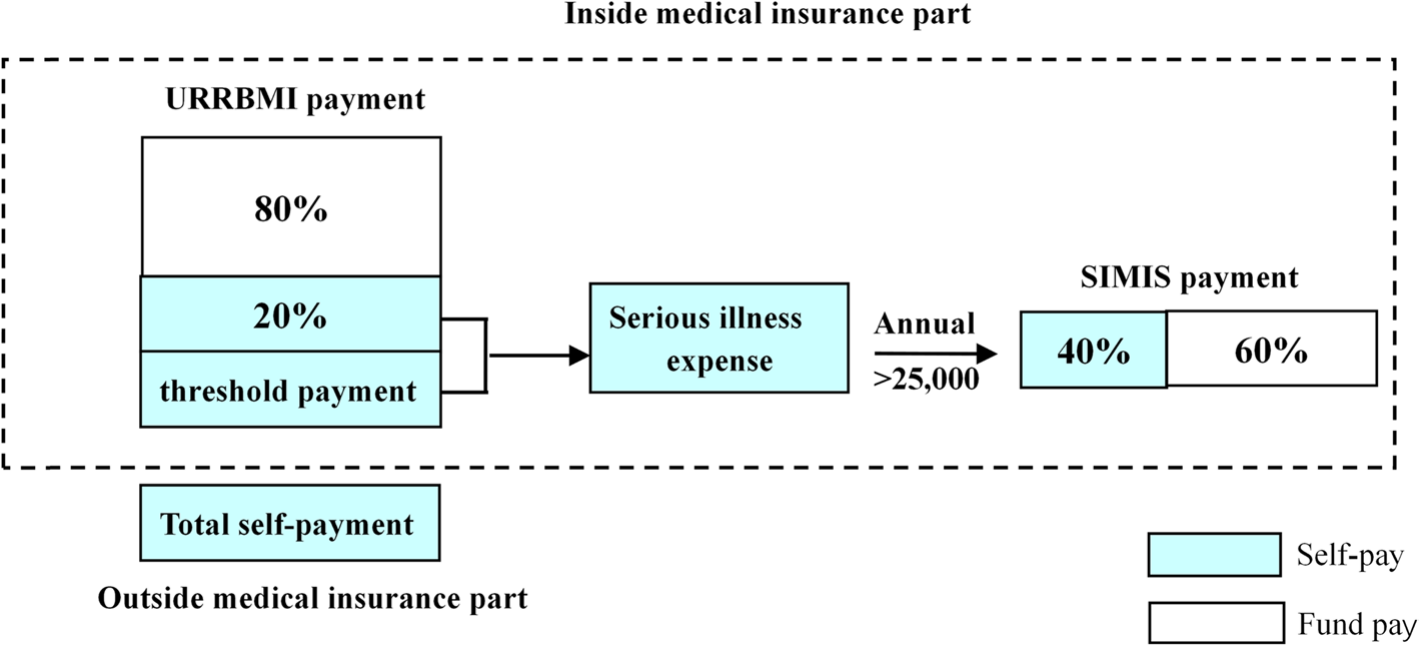
The designed payment scheme of SIMIS in Shanghai

SIMIS is a secondary payment based on basic medical insurance. The payment mode is illustrated in Fig. 1, where total medical expenses can be divided into two parts: inside and outside medical insurance.

#### Inside medical insurance

##### URRMBI payment

[10] According to the policy, total expenses for an inpatient are split into two tiers: total self-payment and URRBMI payments. The URRBMI payment includes the threshold to trigger the URRBMI fund, self-payment under the URRBMI, and URRBMI fund payments.

##### SIMIS payment

[10] The expenses described by SIMIS are called serious illness expenses. The SIMIS payment comes from self-payment under the URRBMI and threshold payment. If the annual serious illness expenses of patients exceed 25 000 yuan, the SIMIS fund is paid proportionally without a ceiling limit; if not, the reimbursement scheme falls under URRBMI payments.

#### Outside medical insurance

##### Total self-payment

[10] Expenses outside the medical insurance system are called total self-payments, which the medical insurance cannot reimburse.

### Construct micro-simulation model

**Fig. 2:**
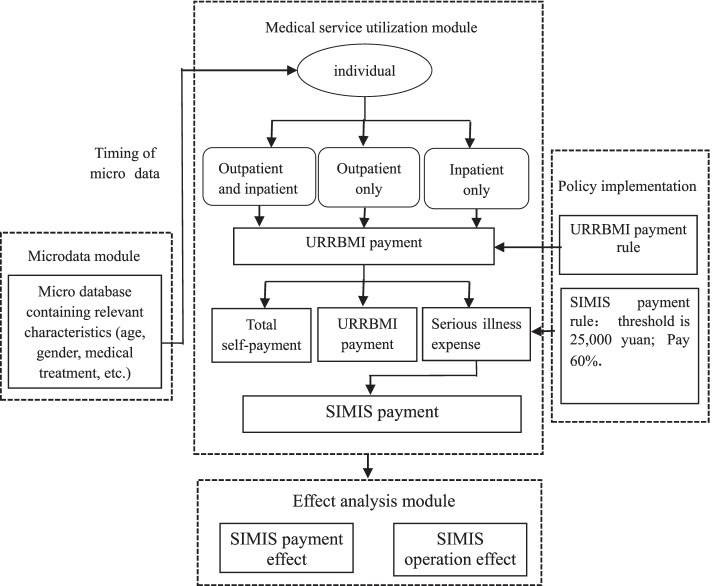
The micro-simulation model of SIMIS

The micro-simulation model (Fig. 2) was constructed according to the designed SIMIS payment system, which was divided into four modules: micro-data, medical service utilisation, policy implementation, and effect analysis module [11–14].

The micro-data module was mainly a micro database obtained by a random sampling of 2% of the population with basic medical insurance in Shanghai. To ensure the accuracy of the analysis, this study focused on the medical treatment of patients with serious illness expenses of more than 10 000 yuan, according to the designed serious illness insurance payment scheme, the residents on both sides of the starting line are the focus of the analysis. Taking the sample in 2016 as an example, when the starting line is 15000 yuan, the number of residents of 10,000–15,000 yuan accounts for 40.76% of the total sample, which can meet the requirements of the analysis quantity. The micro database provided the following information: 1) the distribution parameters of the admission rate by age and sex, 2) the distribution parameters of each patient visit type, and 3) the annual medical consumption growth rate parameters and medical insurance payment proportion parameters for individual patients. The micro database was updated to 2025.

The effect analysis module mainly analyzed the effect of policy implementation by summarising the individual medical consumption and SIMIS results for each target year.

#### Simulation process

The threshold to trigger the designed SIMIS fund is an annual serious illness expense exceeding 25 000 yuan that will be paid again. Therefore, the payment categories can be divided into three: only outpatient medical services (OOMS), only inpatient medical services (OIMS), and both outpatient and inpatient medical services (BOIMS).

#### Insured population of SIMIS estimation

The target population for SIMIS is the population that does not participate in UEBMI. The estimation process was conducted as follows. (1) Based on the total registered residence population published in Shanghai Statistical Yearbook in 2010–2019 [15], the size of the population with registered residency in Shanghai in 2020–2025 is estimated by fitting an exponential curve. Based on the changing trend in the registered resident population in different age categories, the number of registered residents in Shanghai in 2020–2025 years is estimated. (2) Based on the changes in the composition of the insured population by type and age group [16], it is possible to estimate the target insured population with serious illness insurance before 2025. (3) When the participation rate of basic medical insurance in Shanghai is 97%, the actual participation in URRBMI can be estimated. Table 1 shows the estimation results for the insured SIMIS population.

#### Population estimation of seriously ill patients

We summarise the total medical expenses, URRBMI fund payment expenses, self-payment under the URRBMI expenses, and total self-payment expenses of the insured in each year according to the ID code of the insured. If the total amount of serious illness expenses exceeds 10 000 yuan, they are screened and the admission rate is calculated. We find the individual consumption data in outpatients and inpatients according to the patient’s ID code, and summarise the number of patients admitted to OOMS, OIMS, and BOIMS from 2011 to 2016 by age and gender, and determine the distribution of patient admission types. During the simulation, the patients in the current year are determined in combination with the distribution of patient admission rates and admission types.

##### Estimation of admission rate

The actual data show that the admission rate of patients generally increases by a certain amount each year. Therefore, when constructing the admission rate of patients in all the forecast years, a small increase is assumed; for example, rate2017 = rate2016 + (the sum of the added value of admission rate in each year from 2011 to 2015) / 5. The estimated results of the admission rates are given in Table 2.

**Table 1:** Estimation results of insured population of SIMIS

Year	Insured persons	Uninsured persons
2016	3,370,900	435,000
2017	3,438,500	436,900
2018	3,420,400	439,100
2019	3,486,800	441,300
2020	3,569,900	443,100
2021	3,539,700	444,900
2022	3,516,400	446,600
2023	3,593,800	448,300
2024	3,675,000	450,000
2025	3,648,600	451,700

**Table 2:** Parameter estimation of patient admission rate from 2011 to 2025 (%)

Gender	Age	2011	2012	2013	2014	2015	2016	2017	2025
1	1	0.1691	0.1782	0.2128	0.1937	0.6576	0.6785	0.6993	0.8455
1	2	5.5039	6.7469	7.1574	9.7208	7.3669	7.4407	7.5145	8.0313
2	1	0.0604	0.1097	0.1401	0.1916	0.4497	0.4653	0.4809	0.5901
2	2	8.4681	8.0847	16.3417	11.8208	7.6279	7.6641	7.7002	7.9532

Note: 1 = male, 2 = female; age 1 < 60 years, age 2 ≥ 60 years

##### Estimation of admission-type distribution

As there are different types of admissions, patients need to bear different payment percentages of medical service expenses. According to the actual data from 2011 to 2016, the proportion of BOIMS increased slightly, the proportion of OIMS decreased slightly, and the proportion of OOMS increased slightly. In 2017, for example (Table 3), the annual visit types of male patients under the age of 60 were OOMS admission, OIMS admission, and BOIMS admission, with the possibility of 0.113 991, 0.122 510, and 0.763 499, respectively.

**Table 3:** Distribution of visit types in 2016 and 2017

Gender	Age	A2016	B2016	C2016	A2017	B2017	C2017
1	1	0.115 207	0.124 424	0.760 369	0.113 991	0.122 510	0.763 499
1	2	0.063 366	0.009 901	0.926 733	0.063 580	0.009 231	0.927 189
2	1	0.133 333	0.180 000	0.686 667	0.128 784	0.178 859	0.692 357
2	2	0.036 585	0.006 098	0.957 317	0.036 808	0.005 730	0.957 462

Note: 1 = male, 2 = female; age 1 < 60 years, Age 2 ≥ 60 years; A is OOMS admission, B is OIMS admission, and C is BOIMS admission

*OOMS*  only outpatient medical services, *OIMS*  only inpatient medical services, *BOIMS*   both outpatient and inpatient medical services

### Simulation of medical expenses of BOIMS with serious diseases

#### Total medical expense forecast

The model is based on the level of annual medical expenses (4 groups), gender (2 groups, male = 1; female = 2), age (2 groups, 0–59 years = 1; 60 + years = 2). The influencing factors are classified into 16 groups. The annual average medical expenses for each group are calculated. Since the data from 2013 to 2016 are relatively complete, the average value of the growth rate of medical expenses in 2013–2014, 2014–2015, and 2015–2016 is taken as the estimated growth rate of medical expenses in 2017. Using the growth rate of medical expenses and the distribution of patients’ medical expenses in 2016, we calculate the distribution of patients’ medical expenses in 2017. Using the same method, the distribution of patient medical expenses from 2018 to 2025 is obtained. Table 4 lists some of the estimated results.

**Table 4:** Growth rate and average cost estimation of BOIMS medical expenses in 2017

Gender	Age	Expense	Corresponding frequency	Average cost in 2016	Growth rate	Average cost in 2017
1	1	1	0.00—	33,194.15	0.011 19	33,565.75
1	1	2	0.25—	45,858.05	0.018 05	46,685.56
1	1	3	0.50—	62,357.56	-0.012 79	61,560.03
1	1	4	0.75—	138,356.19	-0.010 01	136,970.70
1	2	1	0.00—	38,228.55	0.017 82	38,909.90
2	2	4	0.75—	165,315.70	-0.013 38	163,103.52

Note: The corresponding frequency represents the relative frequency of the four medical cost indicators, from low to high

*BOIMS*  both outpatient and inpatient medical services

When these parameters are used to predict patients’ medical expenses, every seriously ill patient is assigned two uniform random numbers, ran01 and ran02. For example, if a 65-year-old male patient enters the hospital in 2017 and the uniform random number is 0.00 ≤ ran01 < 0.25, the total annual medical costs in the hospital is estimated to be cost17 = (ran02 + 0.5) × 38 909.90 yuan. The random number ran02 is the dispersion of increasing the estimated cost of the same unit, and the number 0.5 ensures that the average cost of this unit is 38 909.90 yuan.

#### Simulation of URRBMI fund payment and serious illness expenses

Before the payment of SIMIS, total personal medical expenses are divided into three parts: the URRBMI fund payment expenses, the serious illness expenses, and total self-payment expenses. For the simulation of the URRBMI fund payment, according to the BOIMS consumption data from 2011 to 2016, the proportion of URRBMI fund payments in the total medical expenses is analysed. The number of classification factors is consistent with the analysis of total expenses. Based on the actual data, the payment proportion in 2014–2016 is relatively stable, so the average proportion in 2014–2016 is taken as the proportion parameter of URRBMI fund payment expenses in 2017–2025. Some of the estimates are presented in Table 5. The proportion of serious illness expense estimations is consistent with that of the URRBMI fund payment expenses.

**Table 5:** Proportion of URRBMI fund payment in total medical expenses from 2014 to 2016

Gender	Age	Expense	Corresponding frequency	2014	2015	2016	Means
1	1	1	0.00—	0.5270	0.5785	0.5660	0.5572
1	1	2	0.25—	0.5377	0.5160	0.5822	0.5453
1	1	3	0.50—	0.5438	0.5454	0.5663	0.5518
1	1	4	0.75—	0.5781	0.4848	0.4924	0.5184
1	2	1	0.00—	0.6142	0.6335	0.6435	0.6304
2	2	4	0.75—	0.5750	0.6334	0.5848	0.5978

Note: Simulation of medical expenses of OOMS and OIMS with serious diseases, where *OOMS*  only outpatient medical services, and *OIMS*  only inpatient medical services

*URRBMI *  urban and rural residents’ basic medical insurance

For the simulation of two types of medical treatment of seriously ill patients, OOMS and OIMS, the process is consistent with the simulation of BOIMS. (1) The number of patients is determined based on the distribution of visit types. (2) The growth trend of total medical expenses in each group from 2011 to 2016 is analysed to obtain the distribution of the growth rate of total medical expenses. (3) Using the data from 2011 to 2016, the proportional distribution of URRBMI fund payment expenses and serious illness expenses in the total medical expenses are determined to simulate all kinds of medical expenses. 3) The simulation focuses on the medical behaviour of patients whose annual serious illness expenses are more than 10 000 yuan.

## Results

When the participation rate of basic medical insurance in Shanghai is 97%, the main simulation results are summarised to analyse the policy effect. Table 6 shows the simulation results of the annual average medical expenses of seriously ill patients from 2020 to 2025.

**Table 6:** Annual per capita medical expenses of seriously ill patients of various medical types

Year	Number of insured persons	Number of visits	Proportion of visits (%)	Per capita cost	Std	Maximum	minimum
OOMS							
2020	3,569,949	1230	0.0345	97,883	18,923	129,259	57,466
2021	3,539,690	1523	0.0430	93,226	24,974	135,021	57,576
2022	3,516,412	1537	0.0437	96,149	26,645	141,059	58,818
2023	3,593,762	1571	0.0437	99,229	28,424	147,347	60,088
2024	3,674,969	1604	0.0436	102,317	30,146	153,915	61,384
2025	3,648,586	1945	0.0533	98,461	33,467	160,775	62,709
OIMS							
2020	3,569,949	819	0.0229	173,194	46,779	197,871	57,195
2021	3,539,690	1279	0.0361	138,059	68,014	209,616	60,790
2022	3,516,412	1401	0.0398	137,222	70,560	222,059	64,610
2023	3,593,762	1512	0.0421	139,710	73,580	235,240	68,671
2024	3,674,969	1575	0.0429	144,303	76,862	249,204	70,950
2025	3,648,586	1160	0.0318	124,833	82,382	263,997	71,369
BOIMS							
2020	3,569,949	23,218	0.6504	137,317	31,795	187,985	80,436
2021	3,539,690	25,405	0.7177	133,027	32,093	187,551	79,487
2022	3,516,412	26,321	0.7485	132,572	31,117	187,118	78,550
2023	3,593,762	26,863	0.7475	132,371	30,269	186,686	77,624
2024	3,674,969	23,940	0.6514	132,318	24,060	156,894	98,159
2025	3,648,586	24,070	0.6597	132,157	22,761	156,748	79,516

Note: *OOMS*   only outpatient medical services, *OIMS*   only inpatient medical services, *BOIMS*   both outpatient and inpatient medical services

As can be seen from Table 7, the total medical expenses of seriously ill patients show an increasing trend, with an average annual growth rate of 3.56%. The URRBMI fund payment covers 56%–58% of total medical expenses, and the SIMIS fund covers 5%–7% of the total medical expenses. Both cover 62%–63% of total medical expenses. Self-payment under SIMIS covers 22%–23% of the total medical expenses, total self-payment covers 14%–15% of the total medical expenses, and the medical expenses borne by individuals cover 36%–38% of the total medical expenses.

**Table 7:** Various payments in total medical expenses

Year	URRBMI fund payment	SIMIS fund payment	Self-payment under SIMIS	Total self-payment	Total medical expense
Payment amount (100 million yuan)					
2020	19.47	2.13	7.74	5.16	34.50
2021	20.94	2.16	8.49	5.40	36.98
2022	21.70	2.23	8.80	5.57	38.29
2023	22.18	2.32	9.03	5.70	39.23
2024	20.30	1.86	8.01	5.26	35.43
2025	20.31	1.76	7.97	5.13	35.17
Payment proportion (%)					
2020	56.41	6.19	22.43	14.97	100.00
2021	56.62	5.83	22.95	14.59	100.00
2022	56.66	5.82	22.98	14.54	100.00
2023	56.53	5.91	23.03	14.53	100.00
2024	57.30	5.26	22.60	14.85	100.00
2025	57.73	5.01	22.66	14.60	100.00

Note: *URRBMI*   urban and rural residents’ basic medical insurance, *SIMIS*   serious illness insurance system

As can be seen from Table 8, in 2025 the maximum payment of SIMIS will be 47 449 yuan and the minimum payment will be 2.09 yuan. The results show that the sense of acquisition is not high for seriously ill patients who have just met the threshold payment. When payments are made based on large expenses, the actual burden of individuals significantly exceeds the per capita disposable income of rural residents, indicating that when serious illness insurance is paid according to high expenses, the poverty reduction effect is not obvious.

**Table 8:** Per capita payment of SIMIS

Year	Number of beneficiaries	SIMIS payment	Self-payment under SIMIS	Maximum payment of SIMIS	Minimum payment of SIMIS
2020	25,267	8449	51,072	33,090	0.02
2021	28,207	7641	49,227	34,585	0.49
2022	29,259	7621	49,106	37,529	0.17
2023	29,946	7749	49,201	40,647	58.36
2024	27,056	6886	49,036	41,546	61.32
2025	27,175	6489	48,220	47,449	2.09

Note: *SIMIS*  serious illness insurance system

## Discussion

Unlike other places, the current SIMIS policy in Shanghai is to pay according to the treatment of diseases after the payment of URRBMI, with no threshold and ceiling. The scope includes dialysis treatment for severe uraemia, anti-rejection treatment in renal transplants, the partial treatment of malignant tumours, and the partial treatment of mental diseases, with a payment proportion of 60% [1]. Relevant research shows that SIMIS pays for treatment according to disease type, with a good cost control effect and higher accuracy, but it cannot pay for high medical expenses outside the scope of protection; SIMIS payment is based on large expenses and covers a wider range of diseases [1, 17, 18]. As long as it exceeds the threshold, patients can obtain payment compensation, but its cost control effect is limited and the guarantee accuracy is insufficient.

From the perspective of the sustainability of the SIMIS fund, in 2020, when SIMIS is paid according to large expenses, the simulation results show that there are 25 000 beneficiaries of serious illness insurance and the fund expenditure is 213 million yuan. When paying according to the current disease treatment, there are 18 000 beneficiaries of SIMIS and the fund expenditure is approximately 126 million yuan. The financing amount of SIMIS funds is 190 million yuan [17–20], indicating that according to the current financing standard, the SIMIS fund pays according to the treatment of diseases, has a balance, and can maintain the normal operation of the fund.

From the aspect of serious illness insurance payments, the simulation results show that the actual payment proportion of SIMIS remains between 5 and 7% when the SIMIS is paid according to large expenses. The proportion of the total medical expenses covered by the payment amount of SIMIS is lower than the amount covered by self-payment in SIMIS and total self-payment, which indicates that the payment intensity of the designed scheme is insufficient. When paying for SIMIS according to the current treatment of serious diseases, the actual payment proportion of SIMIS is about 20%, and the overall payment proportion of medical insurance is about 80% [18], indicating that the payment intensity of SIMIS according to the current policy is higher.

In terms of the poverty reduction effect of serious illness insurance, after the payment of SIMIS, the simulation results show that the average annual cost borne by individual patients ranges from 40 000 to 60 000 yuan, which is higher than the per capita disposable income of rural households, indicating that the medical expenses borne by individuals are still high after the SIMIS payment, and the poverty reduction effect of the designed SIMIS policy is not obvious. After SIMIS is paid according to the current policy, the per capita burden is about 8 000 yuan [18], indicating that the personal burden is relatively low after payment according to disease treatment.

From the application effect of micro-simulation technology on the premise of high quality and the quantity of micro database data, micro-simulation technology has a better evaluation effect on the short-term effect after policy adjustment by mining database information and fully considering the heterogeneity characteristics of seriously ill patients. However, the whole simulation process requires a basic micro database of a high quality, as the quality of the data can affect the model simulation results.The deficiency of this study is that it does not consider the impact of relevant policies and environmental changes on patients' medical behavior. At the same time, because there are few patients with high cost, only the medical characteristics of different ages and genders are considered in the analysis of patients' medical behavior. Next, we will further use relevant theories to analyze the impact of environmental and policy changes on patients' medical behavior.

## Conclusion

SIMIS can further alleviate the economic burden of patients with serious illnesses. Compared with the SIMIS policy scheme required by the national guidance, the current SIMIS payment according to disease treatment in Shanghai is better. The SIMIS fund can maintain normal operation, has certain sustainability, and can further improve the payment proportion of SIMIS. However, we should focus on the economic burden of patients with high expenses outside the scope of payment, and further improve the policy on this basis. Under the framework of the national multi-level medical security system, the positioning of SIMIS should be clearer, which can effectively reduce the risk of serious illness patients returning to poverty due to illness.

## Data Availability

The data used in this study were authorised by the Shanghai Medical Insurance Bureau. The authors also signed a confidentiality agreement with the Shanghai Medical Insurance Bureau. Source data cannot be publicly used. All data generated or analysed during this study are included in this published article and its supplementary information files.

## Abbreviations

UEBMI: Urban Employees Basic Medical Insurance
URRBMI: Urban and Rural Residents Basic Medical Insurance
SIMIS: Serious Illness Medical Insurance System
OOMS: Only Outpatient Medical Services
OIMS: Only Inpatient Medical Services
BOIMS: Both Outpatient and Inpatient Medical Services
